# Safety and Efficacy of Fixed-Dose Combination of Adapalene and Benzoyl Peroxide in Acne Vulgaris Treatment: A Systematic Review of Clinical Trials

**DOI:** 10.7759/cureus.69341

**Published:** 2024-09-13

**Authors:** Komal Sattar, Syeda Sakina, Sarosh Mumtaz, Faiza Behram, Amna Akbar, Sarosh Khan Jadoon, Sabahat Tasneem

**Affiliations:** 1 Dermatology, Russells Hall Hospital, Dudley, GBR; 2 Dermatology, Dr. Sakina Clinics, Wah Cantt, PAK; 3 Dermatology, Combined Military Hospital, Muzaffarabad, PAK; 4 Dermatology, Abbas Institute of Medical Sciences, Muzaffarabad, PAK; 5 Surgery, District Headquarter Hospital, Jhelum Valley, Muzaffarabad, PAK; 6 General Surgery, Combined Military Hospital, Muzaffarabad, PAK; 7 Public Health, Health Services Academy, Islamabad, PAK

**Keywords:** acne, adapalene/benzoyl peroxide, efficacy, randomized controlled trial, safety

## Abstract

The Global Burden of Disease Study 2020 ranked acne vulgaris as the eighth most common skin condition, with a global estimated prevalence of 9.4%. The clinical presentation of acne vulgaris ranges from comedones to nodules and cysts. The main treatment options for acne are retinoids, antibiotics, and benzoyl peroxide (BPO). This study aimed to evaluate the reported efficacy and safety of the combination treatment with retinoid and benzoyl peroxide.

Three databases (ScienceDirect, Google Scholar, and PubMed) were searched using keywords such as acne, adapalene/benzoyl peroxide, and randomized controlled trial, and specific search moods such as "Title and abstract" for PubMed and "Title, abstract, and keywords" for ScienceDirect. On Google Scholar, the "allintitle" option was utilized. The articles were searched for the years between 2003 and 2023.

The eight trials selected for systematic review reported randomized controlled trials in 4,596 individuals with the first trials being conducted in February 2007 and the last one in May 2022. There were 52.8% females in the trials, and the trials reported a primary reduction of acne lesions (efficacy) from 27.5% to 70.2%. The occurrence of adverse effects was also variable (ranging from 2.7% to 57.9%), but these effects were mild and vanished with time.

The results showed that the combination of adapalene and benzoyl peroxide is a safe and effective treatment.

## Introduction and background

Acne is one of the most common dermatological conditions worldwide. It is caused by changes in keratinization, colonization of hair follicles with *Propionibacterium acnes*, inflammatory response of the skin, and increased sebum production induced by androgens. Acne scarring due to severe acne usually lasts a lifetime and has long-term psychological implications [1]. Acne vulgaris is the eighth most prevalent skin disease worldwide, with an estimated prevalence of 9.4% across all age groups, according to the 2020 Global Burden of Disease Study [2]. Acne prevalence varies by country and by age group; 35% to nearly 100% of teenagers experience acne at some stage of their life [3]. The prevalent conditions associated with acne vulgaris are comedones (blackheads and whiteheads), papules (raised lesions of less than 1 cm), and pustules (papule-like lesions that are inflammatory and filled with pus). Nodules and cysts, erythema, hyperpigmentation, and scarring can be noticed in people with acne. Patients may suffer additional detrimental effects in addition to the discomfort experienced due to acne. It has been discovered that acne negatively impacts a person’s social life, sense of self, and body image [4]. It also frequently co-occurs with psychiatric conditions including anxiety, depression, and suicidal thoughts. Furthermore, there is evidence that acne carries significant financial consequences; treating acne in Germany can cost up to 400 million euros a year, and in the US, it can cost nearly 3 billion dollars [5,6].

Multiple researchers looked at the relationship between the presence of acne or severity and several risk variables, many of them being significant like family history (pooled OR=2.91), gender (pooled OR=1.07), and body mass index (pooled OR=2.36). Diet and smoking association were less obvious, and the results of the studies themselves were inconsistent [7]. The management of acne is mainly focused on providing a targeted therapy for Propionibacterium acne. The regulation of sebocyte activity through a range of cellular complex pathways suggests a complex pathogenesis of acne. The study of cellular pathways and hormones and the influence of diet on acne has opened new gateways for research on acne treatment. Propionibacterium acne biofilm formation and antibiotic resistance emphasize the need for new options for acne management [8].

The main treatment options for acne are retinoids, antibiotics, and benzoyl peroxide (BPO). Intolerance to drugs and poor adherence to treatment are the challenges faced at the dermatology clinic. The combination of retinoids and BPO is safe and effective and does not contribute to antibiotic resistance [9]. Adapalene, tazarotene, and tretinoin are the three topical retinoids that the Food and Drug Administration (FDA) has currently approved. Treatment for acne vulgaris with topically applied retinoids is both safe and effective and improves the Investigator's Static Global Assessment and Investigator Global Assessment scores. Topical retinoid monotherapy does not have the same efficacy as topical retinoid combination therapy with an antibiotic. Among topical retinoids, adapalene exhibits a better tolerance profile [10]. Besides, adapalene and BPO combination can be helpful to treat acne as Adapalene is more stable and effective in either the presence or absence of light while combined with BPO [11]. 

Additionally, a wide range of novel treatments that target the inflammatory cascade of acne pathogenesis are presently being researched. These treatments include, among others, small molecule inhibitors that target sebaceous glands and enzymes as well as phytochemicals that are sebo-suppressive and anti-inflammatory. A combination of hoover help and metal nano-shells is being used in laser and light therapy to treat acne. As the effects of the microbiome on many organ systems become clearer, probiotics have become more and more prominent in medicine. Research is still being conducted to better understand how specific bacterial strains that produce ammonia can help regulate the inflammatory response of the skin [12].

## Review

Materials and methods

Search Techniques

Three databases (ScienceDirect, Google Scholar, and PubMed) were searched using the keywords acne adapalene/benzoyl peroxide, randomized controlled trial, and some other following keywords using the Boolean operating system and specific search moods such as "Title and abstract" for PubMed and "Title, abstract, and keywords" for ScienceDirect. On Google Scholar, the "allintitle" option was utilized. For the PubMed search, the following MeSH terms were built: ((((("Acne Vulgaris/diagnosis"[MeSH] OR "Acne Vulgaris/drug therapy"[MeSH] OR "Acne Vulgaris/epidemiology"[MeSH] OR "Acne Vulgaris/etiology"[MeSH] OR "Acne Vulgaris/pathology"[MeSH] OR "Acne Vulgaris/therapy"[MeSH])) AND ("Adapalene, Benzoyl Peroxide Drug Combination/administration and dosage"[MeSH] OR "Adapalene, Benzoyl Peroxide Drug Combination/adverse effects"[MeSH] OR "Adapalene, Benzoyl Peroxide Drug Combination/therapeutic use"[MeSH])) AND "Randomized Controlled Trial" [Publication Type]) AND "Benzoyl Peroxide"[MeSH]) AND "Adapalene"[MeSH], acne adapalene/benzoyl peroxide, and randomized controlled trial. A detailed search strategy is mentioned in the Appendices.

Inclusion and Exclusion Criteria

Full-length research articles were targeted as per the inclusion criteria, especially where the articles reported randomized controlled trials, with blinding preferably double-blind studies, and the intervention was the application of adapalene 0.3% or 0.1% combined with 2.5% benzoyl peroxide (A/BPO) topical gel. Other articles such as reviews, systematic reviews, meta-analyses, letters to the editor, editorials, correspondence, case studies, and articles written in non-English language were excluded as per the exclusion criteria.

Extracting Data

Every study that qualified the inclusion and exclusion criteria provided the following data, which was recorded in an MS Excel Spreadsheet (Microsoft Corp., Redmond, WA): first author, year, country, sex ratio, communication media, treatment benefits, treatment concerns, level of satisfaction, age, number of patients, study timeline, summary goal and outcome, quality assessment score, and study type. The systematic review was done manually including the extraction of data.

Evaluating Study Quality

Studies that answered less than 50% of the checklist's questions were deemed low-quality studies, while those that answered between 50% and 70% of the questions were deemed moderate- and high-quality studies, respectively, according to the Joanna Briggs Institute's (JBI) critical appraisal tools [13]. The low-, moderate-, and high-quality studies imply high, moderate, and low risk of bias, respectively [14].

The systematic review rigorously followed the Preferred Reporting Items for Systematic Reviews and Meta-Analyses (PRISMA) guidelines to ensure comprehensive and transparent reporting of the review process [15].

Results

Study Selection

**Figure 1:**
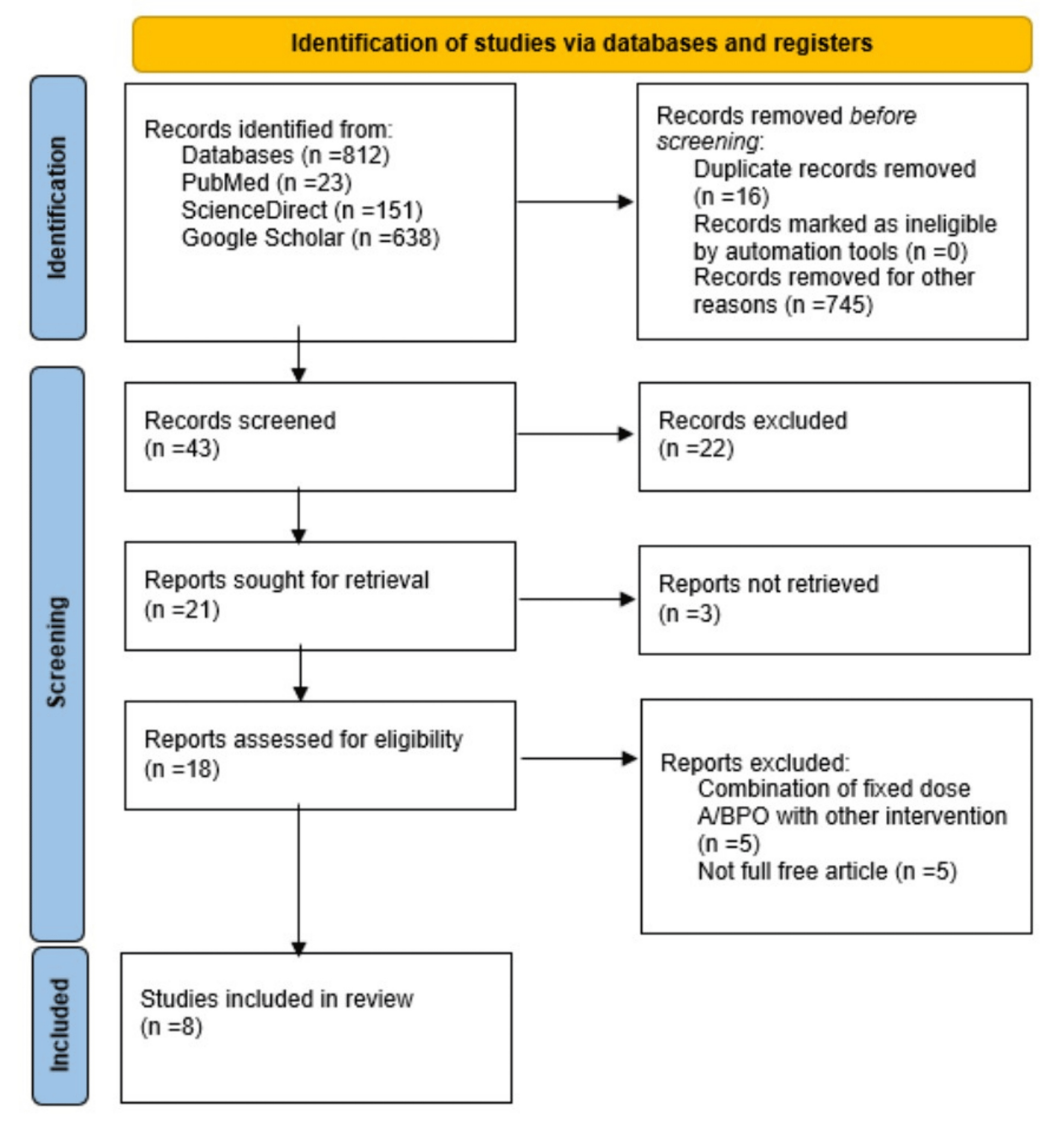
PRISMA flow diagram PRISMA: Preferred Reporting Items for Systematic Reviews and Meta-Analyses, A/BPO: adapalene/benzoyl peroxide

We initially found 812 articles (PubMed: 23, ScienceDirect: 151, and Google Scholar: 638) in total from three databases, and finally, after sequential screening, eight studies were selected for this study. The details are shown in the PRISMA flow diagram (Figure 1).

Acne Scoring

The score for acne severity is as follows: 0, "clear" (erythema and residual hyperpigmentation may be present (complete improvement)); 1, "almost clear" (a couple of straggling comedones and a few little papules); 2, "mild" (less than half of the face is affected and easily identifiable, and certain papules and pustules, along with certain comedones, are present); 3, "moderate" (there is involvement over half the face; numerous pustules, papules, and comedones are present; and there could be just one nodule); and 4, "severe" (there are comedones all over the face, along with many papules and pustules, a few nodules, and cysts (worse)).

Timeline of Studies

We searched articles dated between 2003 and 2023 and obtained published articles dated between 2007 and 2023. The clinical trials were performed from February 2007 [11] to the last trial in May 2022 [16].

Data Extraction

Several data were extracted from the selected studies. The total number of participants in the eight studies was 4,596. The age of the participants was 12 years and older, and the mean age ranged between 16.4 years and 23.4 years. The overall percentage of female gender was 52.87% (2,430/4,596). The studies included were from different locations, such as the United States of America (USA), Puerto Rico, Canada, France, and Europe. Acne type, comparator, criteria for study participant inclusion and exclusion, application type, and total application period were extracted as well.

Drug Applied

The drug used was adapalene 0.1% or 0.3% plus benzoyl peroxide 2.5%. The drug was applied as a once-daily dose.

Comparator

The comparators were vehicle plus monotherapy either with adapalene or benzoyl peroxide [11,17,18], vehicle only [19-22], and retinoid [16].

Outcome Variables

The outcome variables were as follows: primary efficacy (PE); lesion count reduction from baseline (inflammatory lesion (IL), non-inflammatory lesion (NIL), and total lesion (TL) count); success rate; median percentage change in IL, NIL, and total count; subjective assessment; safety; tolerability (adverse events (AEs)); and Scar Cosmesis Assessment and Rating Scale (SCAR-S), Scar Global Assessment (SGA) score, Investigator Global Assessment (IGA) score subjective assessment (skin texture and roughness), retinoid-induced skin discomfort (RISD) composite score, and Global Acne Evaluation (GEA) score.

Quality of Studies

All the studies were high-quality studies (Table 1).

**Table 1:** Quality assessment of the included study Questions: 1. Was the research question appropriate? 2. Was the study population clearly defined? 3. Were controls selected from the same or similar population including the same timeframe? 4. Did the author follow proper inclusion and exclusion criteria regarding case-control selection? 5. Were the cases clearly defined and differentiated from controls? 6. Were the assessors of exposure/risk blinded to the case or control status of participants? 7. Were the methods of quantity determination clearly defined? 8. Did the authors use statistical analyses? 9. Were the measurements/tools valid to conduct the study? Y: yes (score=1), N: no (score=0), P: partially (score=0.5), NR: not reported (no score)

Study ID	1	2	3	4	5	6	7	8	9	Overall score
Thiboutot et al. (2007) [11]	Y	Y	Y	Y	Y	Y	Y	Y	Y	9
Stein Gold et al. (2009) [17]	Y	Y	Y	Y	Y	Y	Y	Y	Y	9
Gollnick et al. (2009) [18]	Y	Y	Y	Y	Y	Y	Y	Y	Y	9
Stein Gold et al. (2016) [19]	Y	Y	Y	Y	Y	Y	Y	Y	Y	9
Dreno et al. (2016) [20]	Y	Y	Y	NR	Y	Y	Y	Y	Y	8
Dreno et al. (2018) [21]	Y	Y	Y	Y	Y	Y	Y	Y	Y	9
Dreno et al. (2019) [22]	Y	Y	Y	Y	Y	Y	Y	Y	Y	9
Khammari et al. (2023) [16]	Y	Y	Y	Y	Y	Y	Y	Y	Y	9

Discussion

Acne is a chronic inflammatory condition affecting the pilosebaceous unit of the skin. There is a dearth of high-quality evidence to support the use of topical or systemic therapies available for acne. The majority of the current recommendations for treating acne are based on professional consensus, and they recommend using topical treatments in mild to moderate cases and systemic medicines in moderate to severe or resistant cases. When topical and general treatments have failed, there is a compelling need for early, efficient treatment with systemic medication due to the psychological effects of acne [1].

Topical treatments include retinoids (e.g., adapalene, isotretinoin (ISO), tazarotene, motretinide, and tretinoin), antibiotics (e.g., erythromycin and clindamycin), and miscellaneous such as benzoyl peroxide, salicylic acid, and azelaic acid. Systemic treatments include retinoids (e.g., isotretinoin), antibiotics (e.g., erythromycin, azithromycin, doxycycline, and clindamycin), and sometimes contraceptives [21]. The last option is physical treatment; some examples include electrocauterization, cryotherapy, extraction of comedones, and intralesional corticosteroids [23]. The present review was meant to discuss the efficacy and safety of the topical benzoyl peroxide (BPO) gel when used in combination with adapalene (A) (Table 2 and Table 3). It also discusses the factors that enhance the effect of the combination (A/BPO) (Table 4).

**Table 2:** Characteristics of the study IGA: Investigator Global Assessment, GEA: Global Acne Evaluation, IL: inflammatory lesion, NIL: non-inflammatory lesion, ISO: isotretinoin, RC: routine care

	Thiboutot et al. (2007) [11]	Stein Gold et al. (2009) [17]	Gollnick et al. (2009) [18]	Stein Gold et al. (2016) [19]	Dreno et al. (2016) [20]	Dreno et al. (2018) [21]	Dreno et al. (2019) [22]	Khammari et al. (2023) [16]
Study type	Randomized, double-blinded, multicenter, parallel-group, controlled trial	Double-blinded, multicenter, randomized, parallel-group, controlled trial	Double-blinded, randomized, multicenter, parallel-group, controlled trial	Double-blinded, multicenter, randomized, parallel-group	Investigator-blinded, multicenter, randomized, vehicle-controlled, international	Investigator-blinded, multicenter, randomized, vehicle-controlled	Randomized, open-labeled extension phase of Dreno et al. (2018)	Double-blinded, randomized, comparative, clinical trial
Location	36 centers in the USA	USA, Puerto Rico, Canada	USA, Canada, and Europe	Canada and USA	Canada and France	Canada	Canada and France	France
Dates	February 17, 2007 to December 21, 2007	2009	2009	July 2013 to March 2014	August 2012 to September 2013	May 2016 to November 2017	NR	September 2021 to May 2022
Time for evaluation	1, 2, 4, and 12 weeks	1, 2, 4, 8, and 12 weeks	1, 2, 4, 8, and 12 weeks	4, 8, and 12 weeks	NR	1, 2, 4, 8, 12, 20, and 24 weeks	24, 36, and 48 weeks	NR
Participants	517	1,668	1,670	503	38	67	45	88
Mean age	16.4 years	18.2 years	19 years	19.3±6.7 years	23.4±3.6 years	21.5 years	21.8 years	21.3±4 years
Gender %	40.2% female	51.3% female	56.2% female	52.3% female	36.8% female	65.7% female	71.1% female	84% female
Acne type	Moderate (80.07%)	IGA 3 (moderate)	IGA 3 (moderate)	Moderate to severe (50/50)	Moderate	Moderate to severe	Moderate to severe	Mild to moderate
Comparator	Vehicle and monotherapy	Vehicle and monotherapy	Vehicle and monotherapy	Vehicle	Vehicle	Vehicle	None	RC
Inclusion	≥12 years age, 20-50 IL and 30-100 NIL, no nodules or cysts	≥12 years age, 20-50 IL, 30-100 NIL, ≤1 nodule, no cyst	≥12 years age, 20-50 IL, 30-100 NIL, ≤1 nodule, no cyst	20-100 IL, 30-150 NIL, ≤2 nodules, no cyst	18-35 years, 20-40 IL, ≤1 nodule, no cyst, ≥10 atrophic scars (≥1.5 mm)	18-35 years, ≥25 IL, ≤2 nodules, ≥10 atrophic scars, IGA 3 and 4	18-35 years, ≥25 IL, ≤2 nodules, ≥10 atrophic scars, IGA 3 and 4	≥16 years age, GEA II and III, ≥15 IL lesions
Exclusion	Pregnancy, nursing, men with facial hair, severe acne requiring ISO	Systemic treatment, acne other than acne vulgaris	Pregnancy, nursing, men with facial hair, severe acne requiring ISO	Systemic treatment, acne other than acne vulgaris, pregnancy	NR	Systemic treatment, acne other than acne vulgaris	Systemic treatment, acne other than acne vulgaris	Pre-menstrual, pregnant, breastfeeding, intolerance to treatment
Application	Once daily	Daily, topical	Once daily	Daily, topical	Daily, split face, topical	Daily, split face, topical	Daily	Daily, split face, topical
Total time	12 weeks	12 weeks	12 weeks	12 weeks	6 months	24 weeks	48 weeks	12 weeks

**Table 3:** Variables measured in each trial and outcomes A/BPO: adapalene/benzoyl peroxide, V: vehicle, A: adapalene, BPO: benzoyl peroxide, IGA: Investigator Global Assessment, GEA: Global Acne Evaluation, SGA: Scar Global Assessment, RISD: retinoid-induced skin discomfort, RC: regular cure, DC: dermo-cosmetic, IL: inflammatory lesion, NIL: non-inflammatory lesion, TL: total lesions, AEs: adverse events, NR: not reported, PE: primary efficacy

	Thiboutot et al. (2007) [11]	Stein Gold et al. (2009) [17]	Gollnick et al. (2009) [18]	Stein Gold et al. (2016) [19]	Dreno et al. (2016) [20]	Dreno et al. (2018) [21]	Dreno et al. (2019) [22]	Khammari et al. (2023) [16]
PE (%)	A/BPO (27.5), A (15.5), BPO (15.4), V (9.9)	A/BPO (70.2), A (60.1), BPO (61.1), V (49.6)	A/BPO (22.9), A (37.3), BPO (40.5), V (45.7)	NR	Acne scar: A/BPO (11.1 to 11.6), V (10.9 to 13.6)	A0.3/BPO2.5 (15.5), V (14.4)	Acne scar: A/BPO (9.2 at 24 weeks), V (13.9 at 12 weeks)	NR
Success rate (%)	A/BPO (30.1), A (18.1), BPO (16.5), V (13.7)	A/BPO (30.1), A (19.8), BPO (22.2), V (11.3)	A/BPO (37.9), A (21.8), BPO (26.7), V (17.9)	Moderate: A0.3/BPO2.5 (33.7), V (11.0); severe: A0.3/BPO2.5 (31.9), V (11.8)	NR	NR	A0.3/BPO2.5 (44.4), A0.3/BPO2.5 + V (28.9)	NR
Acne severity score	IGA score: A/BPO (70.5), A (54.1), BPO (53.7), V (47.9)	NR	IGA score: A/BPO (75.0), A (63.0), BPO (59.0), V (53.0)	NR	SGA count: A/BPO (9.7 to 45.2), V (97 to 6.5)	SGA count: A0.3/BPO2.5 (34.5), V (60.4)	SGA: A0.3/BPO2.5 (48.9 at 24 weeks, 55.6 at 48 weeks), V (24.4 at 24 weeks, 46.7 at 48 weeks)	GEA score: DC 2.75±0.44 versus RC 2.68±0.47
Percentage reduction in acne (%)	NR	IL: A/BPO (62.1), A (50.0), BPO (55.6), V (34.3); NIL: A/BPO (53.8), A (49.1), BPO (44.1), V (29.5)	IL: A/BPO (70.3), A (57.1), BPO (61.9), V (45.5); NIL: A/BPO (62.2), A (50.4), BPO (48.8), V (36.7); TL: A/BPO (65.4), A (52.3), BPO (48.2), V (37.1)	Moderate IL: A0.3/BPO2.5 (27.0), V (14.4); NIL: A0.3/BPO2.5 (40.2), V (18.5); severe IL: A0.3/BPO2.5 (35.17), V (15.46); NIL: A0.3/BPO2.5 (45.61), V (17.25)	IL: A/BPO (72.0), V (40.0); NIL: A/BPO (58.0), V (31.0)	IL: A0.3/BPO2.5 (86.7), V (57.9); NIL: A0.3/BPO2.5 (59.5), V (41.4); TL: A0.3/BPO2.5 (73.3), V (50.9)	IL: A0.3/BPO2.5 (90.7 at 24 weeks, 86.7 at 48 weeks), V (12.8 at 48 weeks); TL: A0.3/BPO2.5 (82.2 at 24 weeks, 77.8 at 48 weeks), V (53.1 at 24 weeks, 74.5 at 48 weeks)	NIL, IL, TL (23.45±6.97, 20.23±4.15, 43.68±9.37 in DC group)
Subjective improvement (%)	A/BPO (42.5), A (34.8), BPO (30.6), V (14.5)	A/BPO (73.5), A (65.6), BPO (66.7), V (55.0)	A/BPO (50.0), A (44.0), BPO (39.0), V (29.0)	A0.3/BPO2.5 (90.7), V (40.0); satisfaction: A0.3/BPO2.5 (71.6), V (32.3)	NR	A0.3/BPO2.5 (64.2), V (19.4); satisfaction: A0.3/BPO2.5 (90.1), V (59.0)	NR	NR
Safety and tolerability (AEs)	1 AE: A/BPO (38.3%), A (42.6%), BPO (29.5%), V (26.8%)	1 AE: A/BPO (2.7%), A (1%), BPO (1.2%), V (0.5%)	1 AE: A/BPO (48%), A (39%), BPO (33%), V (28%)	No irritation: A0.3/BPO2.5 (53%), V (76%)	Total AE: A/BPO (57.9), V (26.3)	A0.3/BPO2.5 (20.9), V (9.0)	NR	RISD: RC 0.84±0.96 versus DC 0.75±0.78

**Table 4:** Interventions or combinations that enhance the action of the combination A/BPO A/BPO: adapalene/benzoyl peroxide, IL: inflammatory lesion, NIL: non-inflammatory lesion, TL: total lesions, AEs: adverse events, NR: not reported, ISO: isotretinoin, IGA: Investigator Global Assessment, SEM: supplementary education material, SCOPE: standard of care patient education, QoL: quality of life, CADI: Cardiff Acne Disability Index, PDRDS: Patient-Doctor Relationship Depth-of-Relationship Scale

	Dreno et al. (2011) [24]	Tan et al. (2014) [25]	Myhill et al. (2017) [26]	Donnarumma et al. (2019) [27]	Stein Gold et al. (2021) [28]
Study type	Double-blind, randomized, controlled, parallel-group	Non-inferiority, investigator-blinded, multicenter, randomized, controlled	Randomized controlled trial, multicenter	Randomized controlled trial, multicenter	Double-blind, multicenter, randomized
Participants	378	266	97	126	741
Age	12-35 years	12-35 years	22.5 years	19.5 years	19.56 years
Gender (%)	44.7% female	NR	69.1% female	68.2% female	61.1% female
Acne type	Moderate to severe	Severe, nodular	Mild to moderate (87.6%)	Mild to severe	Moderate to severe
Combination and comparison	A0.1/BPO2.5 + lymecycline versus vehicle + lymecycline	A0.1/BPO2.5 + doxycycline, comparison with oral isotretinoin	SEM, additional visits, SCOPE	G1: Informative pamphlet, G2: SCOPE + SMS reminder, G3: SCOPE	A/BPO + clindamycin versus vehicle or combination of any two drugs
Inclusion and exclusion	Inclusion: 20 IL, 30-120 NIL, ≤3 nodules; exclusion: acne other than acne vulgaris and pregnancy	Inclusion: age 12-35 years, IGA score ≥ 4, ≥20 papules/pustules, ≥5 nodules	Inclusion: ≥12 years of age, acne grade II and III	Inclusion: 14 and 28 years, IGA 3 and 4, owning a mobile phone capable of receiving text (SMS); exclusion: linguistic or psychiatric issues, non-consent, pregnant or breastfeeding	Inclusion: ≥12 years of age, moderate to severe acne
Application	A0.1/BPO2.5 + oral lymecycline or vehicle + oral lymecycline daily	Oral ISO + vehicle gel, oral doxycycline + A/BPO gel, once daily	Daily, topical	Daily, topical	Once daily
Time	12 weeks	20 weeks	12 weeks	12 weeks	12 weeks
Outcomes	Success rate (47.6% versus 33.7%), lesion reduction (29.9% and 35.5% for IL and NIL) versus 19.6-26.8 and 21.8-30, 70% reduction in total lesion, superior efficacy and well tolerated	A0.1/BPO2.5 + doxycycline had early onset to reduce nodules/pustules and total lesions at two weeks, while ISO was superior in reducing all; 33 adverse events with A/BPO + doxycycline, while 73 AEs with ISO	>75% adherence, SEM more helpful in adherence, use of the drug and management of skin irritation, safety, and physician satisfaction (90%)	According to CADI and PDRDS scores, QoL improvement in SMS and leaflet groups > G3; IGA score reduction was significant in G1 versus G2 and G2 versus G3, but non-significant in G1 versus G3	Success rate (52.5% versus 8.1%), lesion reduction (-74.1% versus -56.8%), well-tolerated combination, satisfaction was more in clindamycin + A/BPO groups

Benzoyl peroxide gel comes in two doses (2.5% and 5%). Both are safe and effective for the long-term treatment of acne vulgaris. The median score for the reduction in inflammatory and non-inflammatory lesion count was 65% on the 12th week and 80% on the 52nd week when these gels were applied in a randomized controlled trial in 458 patients. There were mild to moderate adverse events (AEs) in the first month, but they were transitory and manageable (greater AEs with 5% BPO than 2.5% BPO) [29]. Adapalene is a synthetic topical retinoid that comes in two strengths (0.1% and 0.3%) and is used in a single fixed daily dose with 2.5% benzoyl peroxide. Adapalene has exhibited good tolerability and efficacy in both doses and in combination with BPO as a fixed dose. A0.3/BPO2.5 is used for a range of acne severity and different patient characteristics (age, gender, and ethnicity) for the treatment of moderate to severe acne. A0.3/BPO2.5, when used as a fixed dose daily, offers efficacy and good patient satisfaction by providing flexible dosing instructions. It is safe and tolerable; however, some patients can develop temporary intolerance, which can be managed by patient education and ways to reduce irritation [30]. The combination (A/BPO) not only continues to reduce acne when given over an extended period of six months but also prevents relapse in cases of severe acne. This was proved in a randomized, multicenter, controlled, double-blind study involving 243 subjects having severe acne vulgaris [31].

Treatment Adherence Issue

Discontinuation of treatment causes treatment failures in acne patients mostly due to lack of adherence to treatment. An observation over two months of 1,160 subjects who were receiving five different kinds of treatment showed that the probability of discontinuation was 50%. The reason for discontinuation was adverse effects (AEs) in 9%, ineffectiveness (52%), combination of AEs and ineffectiveness (3%), controlled acne (9%), and other (1%) [32].

Poor adherence to topical treatment for acne is an issue that leads to expenditures and poor quality of life. To improve adherence, supplementary education material (SEM) or extra visits were applied apart from the standard treatment (standard of care patient education (SCOPE)). Subjective assessment was done on patients on a 12-item questionnaire and physicians on a 14-item questionnaire regarding the use of SEM. Safety of treatment was measured in terms of adverse events. Both patients and physicians were satisfied with the SEM along with SCOPE [26]. A randomized trial that combines text message and leaflet services along with A/BPO administration exhibited that this combination is affordable and simple to operate. To improve patient adherence, satisfaction, and treatment outcomes, doctors can think about implementing these procedures in their practices [27]. Another method to enhance adherence is the modulation of the treatment regimen. In a cohort of 120 patients, the A/BPO was either administered daily overnight (A/BPO-EN), for three hours (A/BPO-3h), daily overnight with moisture (A/BPO-moisture), and every other night (A/BPO-EoN). The adherence to treatment, safety, and tolerability of these regimens is improved if implemented for the first four weeks of the treatment [33]. For the treatment of severe acne, isotretinoin (ISO) has been administered orally as a gold standard treatment regimen for the past 30 years. For cases that experience ISO intolerance or side effects and in case of pregnancy, ISO cannot be administered, so an alternative regimen is required. A/BPO + doxycycline carries a favorable profile and can be used in cases of severe and nodular acne [28,25].

There has been a lot of research on the etiology of acne in the past three years that clarified the pathogenesis of acne. The research covers various aspects including sebaceous gland biology, bacteriology, immunology, cell differentiation, sebum production, keratinization, and wound healing. Bacteriology, hyperkeratinization, and inflammation were the targets of previous acne treatments. The biology of the sebaceous glands and immunology are the new aspects that should be explored for acne therapy. The closely linked factors are interleukins (IL-1β, IL-17, and IL-23) and tumor necrosis factor-alpha (TNF-α), offering new therapeutic targets for treating severe acne. The risk factors for developing acne scars include genetics, systematic and local conditions, and social factors. Research has demonstrated that pro-inflammatory cytokines are essential for the formation of hypertrophic scars after acne. It encompasses transforming growth factor-β (TGF-β), IL-6, matrix metalloproteinase (MMP), IGF-1, and B cells. This kind of scar might be easier to avoid and treat in the future if biological antibodies are made that target these cytokines. Future treatments for acne should include methods that focus on the main causes of acne. To reduce the possibility of scarring and other clinical sequelae, such as pigmentary alterations, it would be extremely ideal to place a special emphasis on intensive therapy during the acute inflammatory phase [34]. Studies included in the present review demonstrated that there is good efficacy and tolerability of fixed-dose combination of A/BPO for acne and acne scars. However, the patients included in the trials have mild to moderate or moderate acne, not severe acne. The review emphasizes that the fixed-dose combination can be used in most of the cases but also suggests that the true etiology of acne should be targeted for effective control.

Study limitation

We could not search other databases as we did not have full access to them.

## Conclusions

The results of the studies indicate that the combination therapy of adapalene and benzoyl peroxide (A/BPO) possesses favorable characteristics for acne vulgaris. The primary efficacy of the drug (reduction of lesion count from baseline), reduction in scar count, improved IGA score, and percentage decrease in the lesion count are the outcomes that favor the drug. The drug is also safe as it causes mild to moderate side effects that vanish with time. The drug efficacy can be enhanced by the use of certain interventions such as SMS and literature or by combining with a once-daily dose of an oral drug.

## Appendices

The search strategy used in the present study is shown in Table 5.

**Table 5:** Search strategy MeSH: Medical Subject Headings

Databases	Search strategy
PubMed	((((("Acne Vulgaris/diagnosis"[MeSH] OR "Acne Vulgaris/drug therapy"[MeSH] OR "Acne Vulgaris/epidemiology"[MeSH] OR "Acne Vulgaris/etiology"[MeSH] OR "Acne Vulgaris/pathology"[MeSH] OR "Acne Vulgaris/therapy"[MeSH])) AND ("Adapalene, Benzoyl Peroxide Drug Combination/administration and dosage"[MeSH] OR "Adapalene, Benzoyl Peroxide Drug Combination/adverse effects"[MeSH] OR "Adapalene, Benzoyl Peroxide Drug Combination/therapeutic use"[MeSH])) AND "Randomized Controlled Trial" [Publication Type]) AND "Benzoyl Peroxide"[MeSH]) AND "Adapalene"[MeSH]. acne adapalene/benzoyl peroxide, randomized controlled trial
ScienceDirect	Title, abstract, keywords: Acne Vulgaris therapy randomized controlled trial Title, abstract, keywords: Acne Vulgaris randomized controlled trial Title, abstract, keywords: Acne Vulgaris treatment randomized controlled trial Title, abstract, keywords: Acne Vulgaris drug randomized controlled trial Title, abstract, keywords: Acne Vulgaris drug combination randomized controlled trial
Google Scholar	allintitle: Acne Vulgaris randomized controlled trial allintitle: Acne Vulgaris etiology allintitle: Acne Vulgaris epidemiology allintitle: Acne Vulgaris treatment trial allintitle: Acne Vulgaris therapy trial allintitle: Acne Vulgaris diagnosis allintitle: Acne Vulgaris therapy combination
